# Convolutional neural network based system for fully automatic FLAIR MRI segmentation in multiple sclerosis diagnosis

**DOI:** 10.1038/s41598-025-14112-x

**Published:** 2025-10-16

**Authors:** Ali Arian Darestani, Mahsa Naeeni Davarani, Virginia Guillen -Cañas, Hasan Hashemi, Amin Zarei, Sanaz Heydari Havadaragh, Mohammad Hossein Harirchian

**Affiliations:** 1 Department of Neurosciences, University of the Basque Country (UPV/EHU), Leioa, Spain; 2 Tehran University of Medical Sciences, Tehran, Iran; 3 Biocruces-Bizkaia Health Research Institute, Biocruces, Spain Bizkaia,; 4 Iranian Center of Neurological Research, Neuroscience Institute, Tehran University of Medical Sciences , Tehran, Iran; 5 The Persian Gulf Nuclear Medicine Research Center, Department of Nuclear Medicine, Molecular Imaging, and Theranostics, School of Medicine, Bushehr Medical University Hospital, Bushehr University of Medical Sciences, Bushehr, Iran; 6 Neurology Department, Imam Khomeini Hospital, Tehran University of Medical Sciences, Tehran, Iran; 7 Department of Radiology, School of Medicine, Tehran University of Medical Sciences (TUMS), Tehran, Iran

**Keywords:** Multiple Sclerosis, Convolutional neural networks, FLAIR MRI, Image segmentation, nnU-Net, Neuroscience, Learning and memory, Visual system, Immunological disorders, Neurological disorders

## Abstract

This study presents an automated system using Convolutional Neural Networks (CNNs) for segmenting FLAIR Magnetic Resonance Imaging (MRI) images to aid in the diagnosis of Multiple Sclerosis (MS). The dataset included 103 patients from Imam Khomeini Hospital, Tehran and an additional 10 patients from an external center. Key preprocessing steps included skull stripping, normalization, resizing, segmentation mask processing, entropy-based exclusion, and data augmentation. The nnU-Net architecture tailored for 2D slices was employed and trained using a fivefold cross-validation approach. In the slice-level classification approach, the model achieved 83% accuracy, 100% sensitivity, 75% positive predictive value (PPV), and 99% negative predictive value (NPV) on the internal test set. For the external test set, the accuracy was 76%, sensitivity 100%, PPV 68%, and NPV 100%. Voxel-level segmentation showed a Dice Similarity Coefficient (DSC) of 70% for the internal set and 75% for the external set. The CNN-based system with nnU-Net architecture demonstrated high accuracy and reliability in segmenting MS lesions, highlighting its potential for enhancing clinical decision-making.

**Supplementary Information:**

The online version contains supplementary material available at 10.1038/s41598-025-14112-x.

## Introduction

Multiple Sclerosis (MS) is a chronic neurological disorder where the immune system attacks the central nervous system, causing demyelination and various neurological impairments^1^. The diverse characteristics of MS lesions, including variations in size, shape, and location, create substantial challenges for effective diagnosis and monitoring^2,3^. Magnetic Resonance Imaging (MRI), particularly with Fluid-Attenuated Inversion Recovery (FLAIR) sequences, is crucial for diagnosing and managing MS because of its excellent capability to detect demyelinating lesions in the white matter^4^.

Manual segmentation of MS lesions from FLAIR MRI images is labor-intensive and prone to inter- and intra-observer variability, leading to inconsistent and subjective outcomes^5^. As MS lesions significantly impact patient prognosis and treatment plans, there is a critical need for automated, accurate, and reproducible segmentation methods^6,7^. Recent advancements in artificial intelligence (AI) and machine learning, particularly Convolutional Neural Networks (CNNs), have shown great promise in medical image analysis^8,9^. CNNs excel in feature extraction and pattern recognition from complex datasets, making them highly suitable for MS lesion segmentation. The nnU-Net, a self-adapting neural network framework, represents a state-of-the-art approach in medical image segmentation, offering flexibility and high performance without the need for manual configuration^10,11^.

This study proposes an automated system based on the nnU-Net architecture for segmenting MS lesions in FLAIR MRI images. Our system leverages extensive preprocessing steps, including skull stripping, normalization, resizing, segmentation mask processing, and entropy-based exclusion, combined with advanced data augmentation techniques to enhance model robustness and accuracy. We assembled a dataset comprising FLAIR MRI images from 103 MS patients from Imam Khomeini Hospital in Tehran and an external validation set of 10 patients from another center. The nnU-Net architecture was specifically configured for 2D image slices and trained using a fivefold cross-validation approach on an NVIDIA GeForce RTX 3090 GPU. Performance metrics such as accuracy, sensitivity, positive predictive value (PPV), and negative predictive value (NPV) were employed to evaluate the model on both internal and external test sets.

Our research aims to address the limitations of manual segmentation and enhance the diagnostic workflow for MS. By providing a reliable automated segmentation tool, we seek to facilitate more consistent and precise identification of MS lesions, ultimately improving patient outcomes. The study also explores future directions in distinguishing active from non-active lesions and validating the model on larger, more diverse datasets to ensure its broad applicability.

Our study introduces a fully automatic CNN-based system for segmenting FLAIR MRI images in MS diagnosis, leveraging the nnU-Net architecture for enhanced accuracy and reliability. Unlike previous works that often require manual intervention or are limited by dataset size^12^, our approach demonstrates superior performance on both internal and external test sets, achieving high sensitivity and specificity in lesion detection. This automation and robustness distinguish our method from earlier studies, offering a scalable solution that reduces subjectivity and improves reproducibility in clinical settings.

### Related work

MS is a chronic neurological disorder marked by the immune system attacking the central nervous system, causing demyelination and various neurological impairments. MRI, particularly with FLAIR sequences, is crucial for diagnosing and managing MS due to its capability to detect demyelinating lesions in the white matter^13^. However, manual segmentation of these lesions is labor-intensive and prone to variability, necessitating automated, accurate, and reproducible segmentation methods^14^.

Recent studies have explored various CNN-based methods for segmenting MS lesions in MRI images. For instance, Naeeni Davarani et al.^15^ introduced an efficient approach for the segmentation of active and inactive plaques within FLAIR images using a DeepLabV3Plus SE model with an EfficientNetB0 backbone, demonstrating superior performance compared to other CNN architectures1. This study highlights the potential of advanced deep learning models in improving the accuracy and reliability of MS lesion segmentation.

Advances in AI and machine learning, specifically CNNs, have shown significant promise in medical image analysis^8^. CNNs are particularly adept at feature extraction and pattern recognition in complex datasets, making them well-suited for MS lesion segmentation^16^. Numerous studies have explored CNN-based methods for segmenting MS lesions in MRI images, each demonstrating unique approaches and varying levels of success. Below is a summary of notable research in this field:

In continuation of Table 1, detailed explanations for the summarized studies are provided below:

**Table 1:** The table below summarizes recent studies on MS lesion segmentation using CNNs.

Authors	Datasets	Methods	Limitations	Results
Brown RA et al^17^	Own dataset	FCNN	Agreement with manual segmentation	Dice score: 0.74 (Jacard index)
Coronado I et al^18^	Own dataset	3D CNN	High false-positive rate in small lesions	Dice score: 0.77
Essa E et al^19^	MICCAI 2008 MS challenge dataset	Region-based Convolutional Neural Network (R-CNN)	Need for large annotated datasets	Dice score: 0.83
Birenbaum A et al^20^	2015 Longitudinal MS Lesion Segmentation Challenge	Single View CNN (V-Net) and Longitudinal Network (L-Net)	Performance compared to trained human raters	Dice score: 0.627
Aslani S et al^21^	ISBI 2015, Private dataset	Deep end-to-end 2D CNN	Requires validation on larger datasets	Dice score: 0.6114 (ISBI), 0.6655 (Private)
Nichyporuk et al^22^. (2022)	Clinical trials datasets	Trial-conditioned CIN, naive pooling, single-trial baselines	Handling biases in the label generation process	Dice scores: 0.795,
Wiltgen et al^23^	In-house dataset, MSSEG, ISBI 2015, MICCAI 2008	Ensemble of three 3D UNets	Requires large dataset for training, limited generalizability to unseen data	Dice score: 0.67
Gabr et al^24^	CombiRx clinical trial dataset	FCNN	Variations in class sizes, reliance on multimodal MRI data	Dice scores: 0.95 (WM), 0.96 (GM), 0.99 (CSF), 0.82 (T2 lesions)
Duong et al^25^	Hospital of the University of Pennsylvania	3D U-Net CNN	Variability in lesion characteristics and acquisition parameters	Dice score: 0.789,
Afzal et al^26^	ISBI, MICCAI datasets	Cascaded 2D CNNs	Overlapping lesions, lesions near cortex	Dice scores: ISBI: 0.67, MICCAI: 0.72
de Oliveira et al^27^	ISBI 2015, In-house dataset	FCNN	Limited test group size, need for larger validation	–

It includes the purpose, datasets, methods, limitations, and key results of each study, highlighting advancements and effective approaches in this field.

Brown RA et al^17^ aimed to automatically segment orbital fat to remove technical intensity artifacts using a Fully Convolutional Neural Network (FCNN). The study involved their own dataset and showed good agreement with manual segmentation, achieving a Dice Score of 0.74.

Coronado I et al^18^ focused on the automatic segmentation of gadolinium-enhancing lesions in their own dataset of 1,006 RRMS patients using a 3D CNN. Despite a high false-positive rate in small lesions, the method achieved a Dice Score of 0.77.

Essa E et al^19^ presented a competitive segmentation method for MS lesions using the MICCAI 2008 MS challenge dataset. Their approach, based on a Region-based Convolutional Neural Network (R-CNN), highlighted the need for large annotated datasets and achieved a Dice Score of 0.83.

Birenbaum A et al^20^ proposed an improved MS lesion segmentation method using the 2015 Longitudinal MS Lesion Segmentation Challenge dataset. They employed a Single View CNN (V-Net) and a Longitudinal Network (L-Net), reporting a Dice Score of 0.627.

Aslani S et al^21^ developed a high accuracy MS lesion segmentation technique using the ISBI 2015 and a private dataset of 37 MS patients. Their deep end-to-end 2D CNN with a multi-branch down-sampling path required validation on larger datasets and achieved Dice Scores of 0.6114 (ISBI) and 0.6655 (private).

Nichyporuk et al^22^ addressed the impact of annotation style on medical image segmentation performance using RRMS, SPMS, and PPMS datasets from clinical trials. Their trial-conditioned CIN, naive pooling, and single-trial baselines method handled biases in the label generation process, achieving Dice scores ranging from 0.731 to 0.795 across different conditions.

Wiltgen et al^23^ proposed a deep learning ensemble for accurate MS lesion segmentation using an in-house dataset, MSSEG, ISBI 2015, and MICCAI 2008 datasets. Their ensemble of three 3D UNets with a composite loss function required large datasets for training and demonstrated limited generalizability to unseen data, achieving an overall Dice Score of 0.67 and 0.65 for MSSEG-1.

Gabr et al^24^. (2019) used a Fully Convolutional Neural Network (FCNN) for brain and lesion segmentation in MS patients, utilizing the CombiRx clinical trial dataset. Their approach, which relied on multimodal MRI data, reported Dice Scores of 0.95 (WM), 0.96 (GM), 0.99 (CSF), and 0.82 (T2 lesions).

Duong et al^25^ developed an automated FLAIR lesion segmentation method across multiple pathologies using a 3D U-Net CNN architecture. Evaluated on training and validation cases from the Hospital of the University of Pennsylvania, the method achieved a Dice Score of 0.789 and a correlation with true lesion volume of 0.99.

Afzal et al^26^ focused on the robust segmentation of MS lesions using cascaded CNNs with datasets from ISBI and MICCAI. Their cascaded 2D CNNs method for initial segmentation and false positive reduction addressed overlapping lesions and lesions near the cortex, achieving Dice Scores of 0.67 (ISBI) and 0.72 (MICCAI).

de Oliveira et al^27^ aimed to quantify brain lesions in MS patients using the ISBI 2015 and an in-house dataset. Their method employed FCNN and preprocessing steps such as rigid registration, skull stripping, and bias correction. Despite the limited test group size, their approach contributed to the volume quantification with a test group range of 0.51 × 10^4—5.85 × 10^4 mm^3^.

### Dataset

In this study, we used FLAIR MRI images from multiple sclerosis (MS) patients. Initially, we collected data from 120 patients. Detailed patient information is described in Table 1. However, after a thorough re-evaluation by experts, we excluded some patients due to insufficient information, resulting in a final dataset comprising 103 patients. All images were collected from Imam Khomeini Hospital in Tehran, following the imaging protocol described in Table 2.

**Table 2:** This table summarizes patient demographics and clinical characteristics from the original center and external validation groups, including gender, age distribution, disease type, and treatments received.

	Category	Original center patients count	External validation patients count
Gender Distribution	Female	75	7
	Male	28	3
Age distribution	Number of patients	103	10
	Mean Age	33.07 years	33.7 years
	Standard deviation	10.61	11.1
	Minimum age	16 years	18 years
	Maximum age	64 years	60 years
	25th percentile	24	29
	50th percentile	33	33
	75th percentile	42	44
Disease type	Relapsing–remitting (RR)	68	7
	Secondary progressive (SP)	4	1
	Primary progressive (PP)	1	1
	Missing data	30	1
Treatments	Dimethyl fumarate (DMF)	4	2
	Glatiramer acetate (GA)	6	0
	Rituximab (RTX)	5	6
	Interferon beta-1a (AVONEX)	3	0
	Other Treatments (each)	51	0
	Missing Data	34	2

The FLAIR MRI images were acquired with a resolution of 512 × 512 pixels, using a 1.5 T MRI scanner with a repetition time (TR) of 9000 ms and an echo time (TE) of 120 ms. The slice thickness was 5.5 mm with a 1 mm gap between slices, ensuring sufficient coverage of brain structures critical for MS lesion detection. These acquisition parameters, detailed further in Table 3, were consistently applied across all patients to maintain uniformity in the dataset.

**Table 3:** Imaging protocol was used for MRI acquisition.

Acquisition plane: AXIAL	MR acquisition type: 2D
Receive coil name: BrainArrayII	Field Strength: 1.5 Tesla
Flip Angle: 90 degrees	Manufacturer: GE MEDICAL SYSTEMS
Rows: 512	Pixel Spacing X: 0.42969 mm ~
Columns: 512	Pixel Spacing Y: 0.429688 mm ~
Spacing Between Slices: 6.5 mm	Slice Thickness: 5.5 mm

Additionally, for external testing, we utilized data from two additional centers outside of Imam Khomeini Hospital, comprising an additional 10 patients. This external dataset provided further validation and assessment of our proposed methods and models.. Details of these patients are also described in Table 2.

Table 3 outlines the imaging protocol utilized for MRI acquisition. It includes specific details regarding the imaging plane, acquisition type, receiver coil, field strength, flip angle, manufacturer, number of rows and columns, pixel spacing in both X and Y directions, slice spacing, and slice thickness.

The table illustrates that the axial plane, which is crucial for diagnosing Multiple Sclerosis (MS), was selected as the primary imaging plane for each patient. Each MRI volume was subsequently divided into individual slices within this plane, generating approximately 20–25 slices per patient. Every slice was meticulously examined and labeled by experts using the Pixlr Suite program. The labeling process was validated by a radiologist who ensured the presence of lesions across axial, sagittal, and coronal dimensions. Ground truth masks were provided for slices identified with lesions to facilitate the training and validation of the model.

In total, approximately 1200 slices with ground truth masks were selected from Imam Khomeini Hospital in Tehran. Additionally, data from an external center contributed 23 more slices with ground truth masks, further enriching the dataset and ensuring robust model training and evaluation.

### Preprocessing

**Fig. 1:**
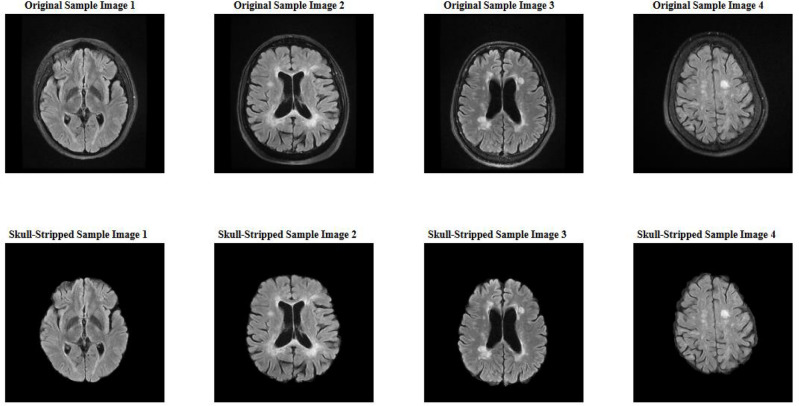
Example of a skull-stripped FLAIR MRI image.

In preparation for training and validating the convolutional neural network (CNN) model, the following preprocessing steps were applied to the FLAIR MRI images and their corresponding segmentation masks:

1. **Skull Stripping:** Each volume of FLAIR MRI images underwent skull stripping using FMRIB Software Library (FSL) (https://fsl.fmrib.ox.ac.uk/). This process removes non-brain tissue, ensuring that subsequent processing focuses only on brain structures relevant to multiple sclerosis (MS) lesion segmentation. An example of a skull-stripped MRI image is shown in Fig. 1.
2. **Normalization:** Grayscale images were normalized to the range [0, 1]. This step ensures uniformity in pixel values, facilitating consistent input data for the CNN.
3. **Resizing:** Images were resized to 512 × 512 pixels to maintain consistent dimensions across the dataset. Standardizing the image size enhances processing efficiency and supports effective feature extraction by the CNN.
4. **Segmentation Mask Processing:** Ground truth segmentation masks were preprocessed to match the dimensions of the resized images (512 × 512 pixels) and ensure alignment with each MRI slice. Additionally, Masks generated from different segmentation tools were standardized into a uniform format: 0 indicates background (no lesion), and 1 indicates the presence of a lesion (foreground), representing both active and non-active plaques of multiple sclerosis (MS). This standardization enables consistent model training and validation across varying segmentation sources.
5. **Entropy Check:** To address errors in the brain extraction algorithm and distortions typically occurring in the initial and final slices of brain-extracted images, we implemented an entropy-based exclusion criterion. Segmentation masks related to these problematic slices often contained minimal lesion content and only a small number of pixels. We calculated the Shannon entropy for each slice’s mask using Eq. (1).
  1 $$H=-{\sum }_{i} {p}_{i} {log}_{2}({p}_{i})$$

Masks with an entropy lower than 0.01 were removed from the dataset. This step ensures the exclusion of images with insufficient lesion information as well as the initial and final slices that often crashed after skull stripping, thereby improving the overall dataset quality.

6. **Data Augmentation:** Data augmentation is employed to enrich the dataset and improve the model’s robustness. Random transformations are applied to each image slice, generating additional variations for training. The augmentation criteria include:

1. Rotation range: ± 40 degrees
2. Width shift range: ± 20% of the image width
3. Height shift range: ± 20% of the image height
4. Shear range: ± 20 degrees
5. Zoom range: ± 20%
6. Horizontal flipping

These transformations generate five new images for each original slice in the dataset, effectively expanding the dataset size. This augmentation strategy introduces variability in MS lesion characteristics, enhancing the CNN’s ability to generalize and improve its performance on unseen data.

These preprocessing steps prepare the dataset effectively for training a CNN model to accurately segment MS lesions in FLAIR MRI images, ensuring robust performance and interpretation.

## Proposed method

**Fig. 2:**
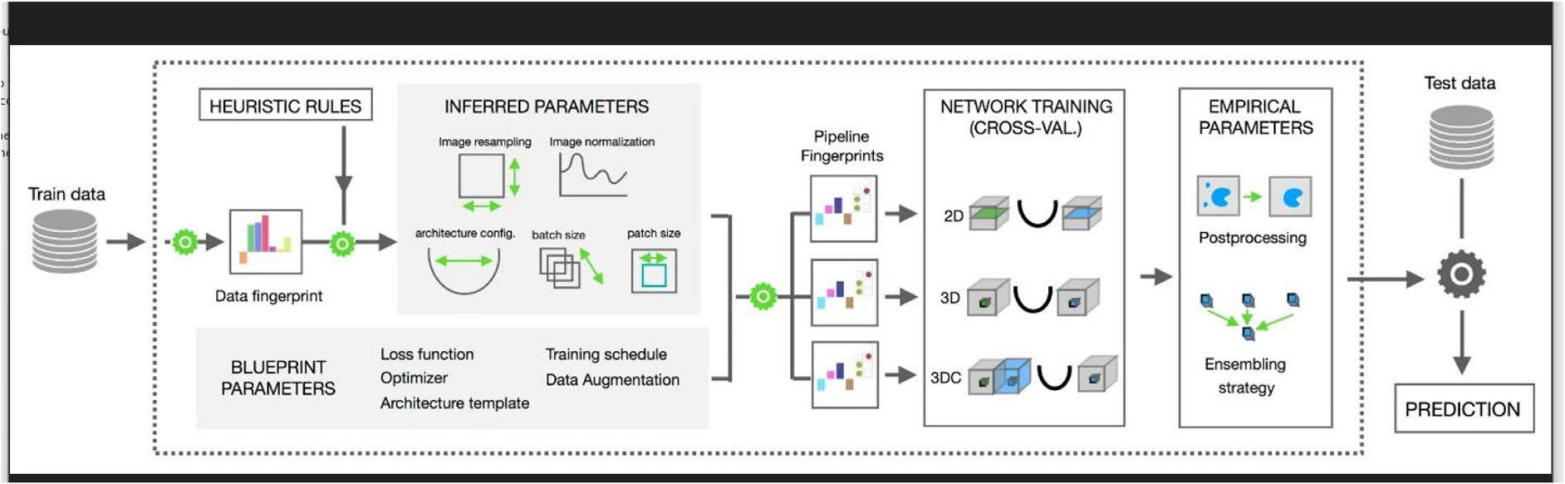
nnU-Net complete workflow.

The proposed method involves a UNet architecture specifically designed for the segmentation of medical images, particularly FLAIR MRI images in the context of multiple sclerosis. The architecture includes:

1. **Network Architecture**: The architecture used in this study is nnU-Net^11^. nnU-Net is an open-source tool that can be effectively used out-of-the-box, rendering state-of-the-art segmentation and catalyzing scientific progress as a framework for automated method design. It provides an end-to-end automated pipeline that can be trained and inferred on any medical dataset for segmentation. Figure 2 illustrates the architecture of nnU-Net^11^.

nnU-Net systematically analyzes the provided training cases and creates a dataset fingerprint, allowing it to automatically configure a matching U-Net-based segmentation pipeline. For each dataset, nnU-Net creates several U-Net configurations:

1. **2D U-Net:** Designed for both 2D and 3D datasets, this configuration processes each slice of 3D images independently.
2. **3D Full Resolution U-Net:** Operates on high-resolution 3D images (for 3D datasets only).
3. **3D Low Resolution → 3D Cascade Full Resolution:** A 3D U-Net cascade where an initial 3D U-Net operates on low-resolution images and a second high-resolution 3D U-Net refines the predictions of the first (for large 3D datasets only).

In our study, we utilized the 2D U-Net configuration because our dataset consists of 2D slices of patient images. This approach allows the model to effectively process and segment each slice independently while leveraging the power of nnU-Net’s automatic adaptation and configuration capabilities.

2. **Hardware Specifications:** Training and validation were performed on an NVIDIA GeForce RTX 3090 GPU with 24 GB VRAM, an Intel Celeron(R) G5905 CPU, 24 GB DDR4 RAM, and a 256 GB SSD for fast data access.
3. **Model Training**: The depth of the network is automatically determined based on the input image size and the desired patch size, ensuring efficient feature extraction^11^. The architecture typically begins with 32 initial filters, doubling with each downsampling layer, and utilizes standard 3×3 convolutional kernels^11^. Downsampling is achieved through 2×2 max pooling operations. The model employs a combination of Dice and binary cross-entropy loss functions with equal weighting to effectively handle class imbalance. Training is conducted using Stochastic Gradient Descent (SGD) with Nesterov momentum set at 0.99 and a weight decay of 3e-5. The initial learning rate is set to 0.01 and follows a polynomial decay schedule throughout training. The network components are trained using 5-fold cross-validation, with each fold trained for a total of 250 epochs^11^. Batch size is determined automatically based on available GPU memory, typically ranging between 2 and 12. The patch size is adaptively set to cover a significant portion of the input image, ensuring efficient training.

4. **Model Evaluation**: The performance of the segmentation pipeline that was developed was evaluated by comparing the voxel-level results of the fully automatic segmentation mask with the manual segmentation of the corresponding internal and external test sets. To ensure a thorough evaluation of the network’s performance, evaluation metrics were calculated at two levels, assessing the model’s ability to perform two different computer vision tasks: slice-level classification and voxel-level segmentation.^28^

1. **Slice-Level Classification:** This refers to the model’s ability to accurately predict whether a slice scan is positive or negative. A positive scan is defined as a scan where at least one MS-avid lesion is detected in the ground truth manual segmentations. To be considered a true positive prediction, the model must detect at least one lesion in a positive scan with a volumetric overlap of at least 10% compared to the ground truth. A true-negative prediction is when the model does not predict any positive voxels in a negative scan. The accuracy, sensitivity, PPV, and negative predictive value (NPV) are used to assess the classification performance.
2. **Voxel-Level Segmentation:** Network segmentation accuracy is evaluated by comparing the automated model output with the ground truth contour at the voxel level. This is quantified using the Dice Similarity Coefficient (DSC), PPV, Intersection over Union (IoU), and sensitivity.

nnU-Net has set a new benchmark in the field of medical image segmentation without the need to fine-tune a new architecture for every dataset individually. The pipeline itself takes care of hyper-parameter tuning and requires no change in the network architecture to achieve state-of-the-art results. This configuration allows for efficient processing of 2D FLAIR MRI slices, ensuring high performance in segmenting multiple sclerosis lesions.

For more details on the nnU-Net design choices and empirical pipeline configurations based on dataset properties, refer to Fabian Isensee et al.^11^ and the associated GitHub repository (https://github.com/ MICDKFZ/nnUNet).

## Results

To evaluate the performance of the trained model, 25% of the images from all patients in the dataset were used as internal test samples. Care was taken to prevent data leakage during the division of the data into train and validation sets. We ensured that the image slices of any patient in the test samples were not present during the training process. Additionally, the validation data used for the fivefold process were separated from the training data and isolated before performing augmentation. Table 4 reports the validation accuracy for the proposed model in each of the 5 folds. The maximum Dice validation score was achieved in fold 2. The mean Dice validation score across the 5 folds was 86.7%, with a standard deviation of 2.54%.

**Table 4:** the validation dice score for the proposed model in each of the 5 folds.

	Fold 1	Fold 2	Fold 3	Fold 4	Fold 5	Mean ± std
Validation DSC	83.5	89.4	88.4	87.5	84.5	86.7 ± 2.54

**Fig. 3:**
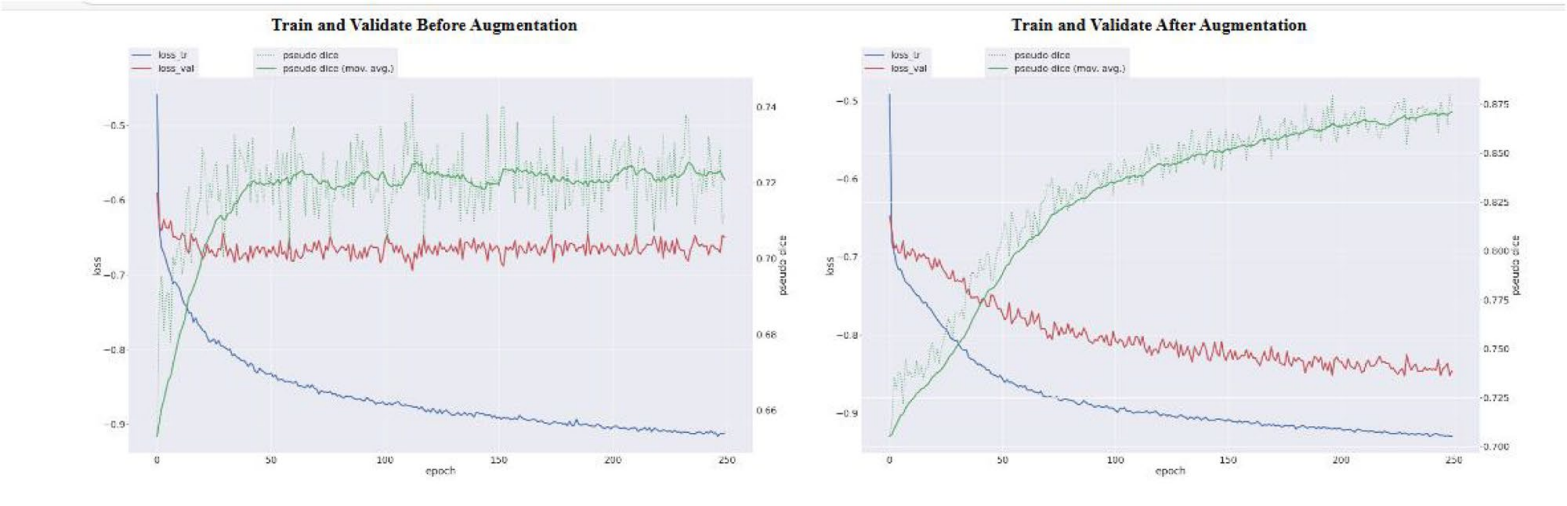
model training and validation performance before(left) and after(right) augmentation.

In Fig. 3, the model’s performance during learning on train and validation samples before and after data augmentation over 250 epochs is shown. As observed, data augmentation resulted in reduced overfitting and improved model performance. Specifically, before data augmentation, the training loss rapidly decreased and reached a low level, while the validation loss plateaued at a higher level, indicating overfitting. However, after applying data augmentation, both training and validation losses decreased more gradually and consistently, resulting in a lower final validation loss. This demonstrates that data augmentation effectively enhanced the model’s generalization ability by introducing variability and preventing the model from overfitting to the training data.

The performance of the model for slice-level classification and voxel-level segmentation is presented in Table 4. The detailed performance metrics calculated on the dedicated test sets from two centers are further summarized in Table 5. To select normal slices, we used slices that specialists did not diagnose any abnormalities in and had no labels. Additionally, to prevent data imbalance during evaluation, normal data were randomly selected in quantities equal to the existing test data and used for the classification process.

**Table 5:** The model performance calculated on the dedicated test sets of two centers.

Task	Metric	Center 1	Center 2
Slice-level classification	Accuracy	83.3% (79.5–86.5%)	76.1% (62.1–86.1%)
	Sensitivity	99.5% (98.3–99.9%)	100% (92.3–100%)
	PPV	75.1% (70.8–78.9%)	67.7% (53.2–79.4%)
	NPV	99.3% (98.0–99.8%)	100% (92.3–100%)
Voxel-level segmentation	DSC	70.3% (68.3–72.3%)	74.8% (70.6–78.9%)
	PPV	75.3% (73.2–77.3%)	88.9% (83.7–94.2%)
	IoU	56.0% (53.9–58.1%)	60.5% (55.6–65.5%)
	Sensitivity	71.1%(68.4–73.8%)	68.1% (60.6–75.5%)
	Hd95	28.3 (23.5–33.1)	31.5 (15.0–48.0)

In terms of slice-level classification, the model achieved an accuracy of 83%, sensitivity of 100%, PPV of 75%, and NPV of 99% for the internal testing set (center 1). Out of the 218 positive scans, the model correctly classified 146 scans as positive, and out of the 218 negative scans, the model correctly classified 217 scans as negative.

For the external testing set (center 2), the model achieved an accuracy of 76%, sensitivity of 100%, PPV of 68%, and NPV of 100%. Out of the 23 positive scans, the model correctly classified 12 scans as positive, and out of the 23 negative scans, the model correctly classified all 23 scans as negative.

**Fig. 4:**
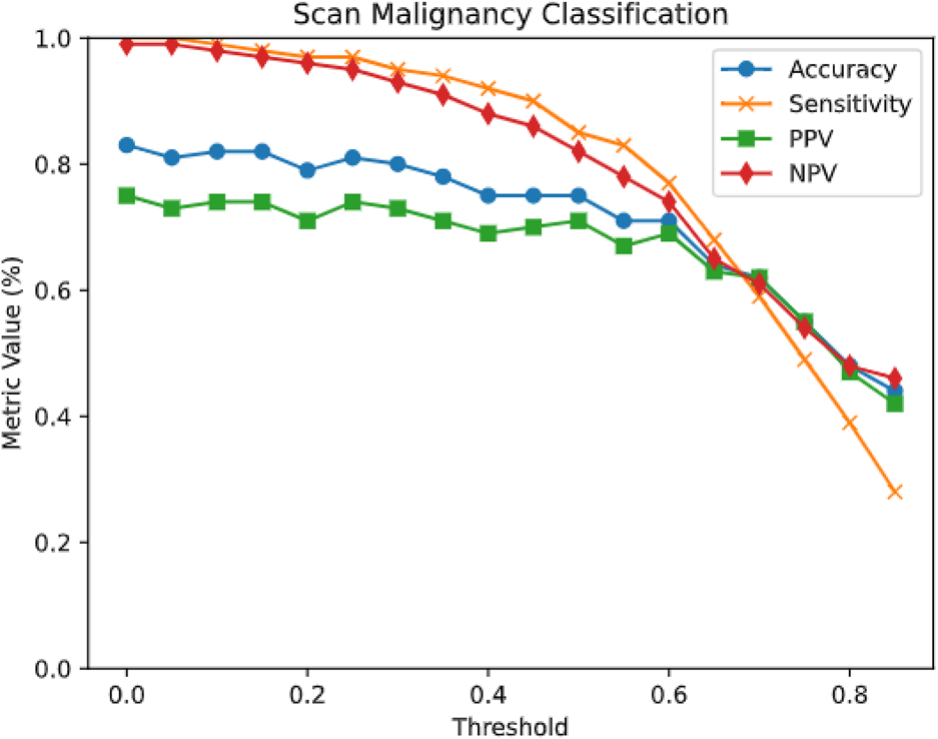
Plots depicting the alteration in calculated metrics as the true-positive threshold is adjusted for the tasks of scan malignancy classification for center 1 (internal testing set).

**Fig. 5:**
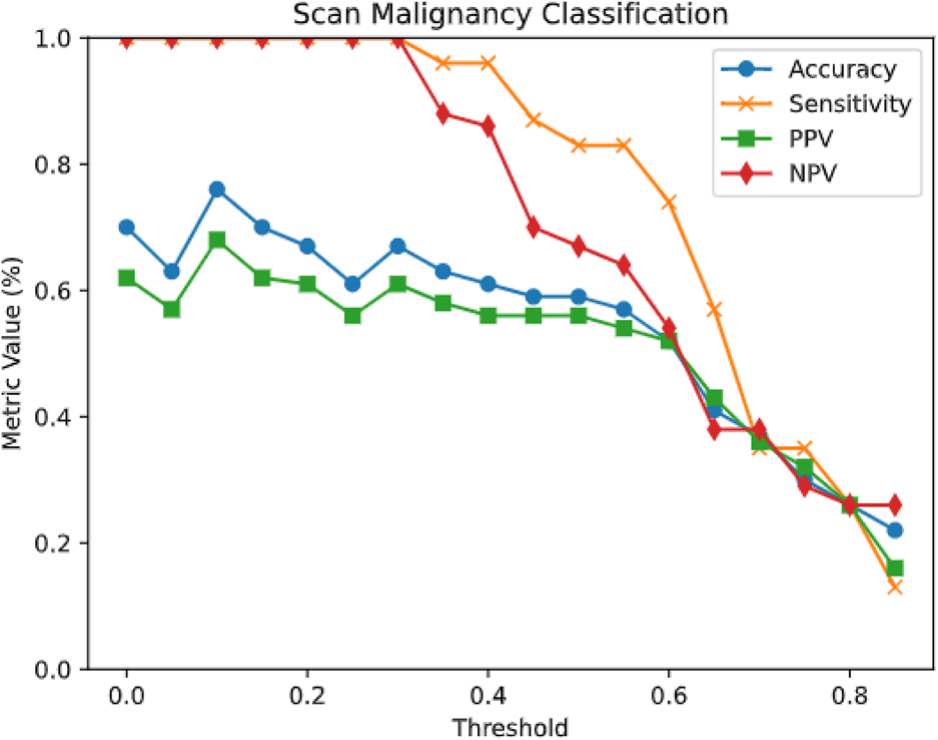
Plots depicting the alteration in calculated metrics as the true-positive threshold is adjusted for the tasks of scan malignancy classification for center 2 (external testing set).

Figures 4 and 5 show the alteration in calculated metrics as the true-positive threshold is adjusted for the tasks of slice-level classification for center 1 (internal testing set) and center 2 (external testing set), respectively.

In terms of voxel-level segmentation, the automated method demonstrated robust performance across both testing centers. For the internal validation set (Center 1), the model achieved a Dice Similarity Coefficient (DSC) of 70.3% (CI 68.3–72.3%), with strong precision (PPV: 75.3% (CI 73.2–77.3%)) and sensitivity (71.1% (CI 68.4–73.8%)). The Intersection over Union (IoU) reached 56.0% (CI 53.9–58.1%), while boundary accuracy measured by HD95 was 28.3 mm (CI 23.5–33.1 mm).

The external validation (Center 2) showed even higher segmentation precision with DSC of 74.8% (CI 70.6–78.9%) and notably improved PPV (88.9% (CI 83.7–94.2%)). While sensitivity remained strong at 68.1% (CI 60.6–75.5%), the IoU improved to 60.5% (CI 55.6–65.5%). The boundary measurement HD95 was slightly higher at 31.5 mm (CI 15.0–48.0 mm), reflecting expected variability in external datasets.

For classification performance, the model maintained excellent accuracy in both centers (Center 1: 83.3% (CI 79.5–86.5%); Center 2: 76.1% (CI 62.1–86.1%)), with perfect sensitivity (99.5% (CI 98.3–99.9%) and 100% (CI 92.3–100%), respectively). The high NPV values (99.3% (CI 98.0–99.8%) and 100% (CI 92.3–100%)) confirm reliable negative case identification.

These comprehensive metrics demonstrate the model’s consistent performance across different clinical environments, with particularly strong precision in external validation (PPV: 88.9% (CI 83.7–94.2%)) and reliable sensitivity in both datasets (> 68%). The narrow confidence intervals for DSC (CI ± 2–4%) indicate stable segmentation performance across various slices and patient cases.

**Fig. 6:**
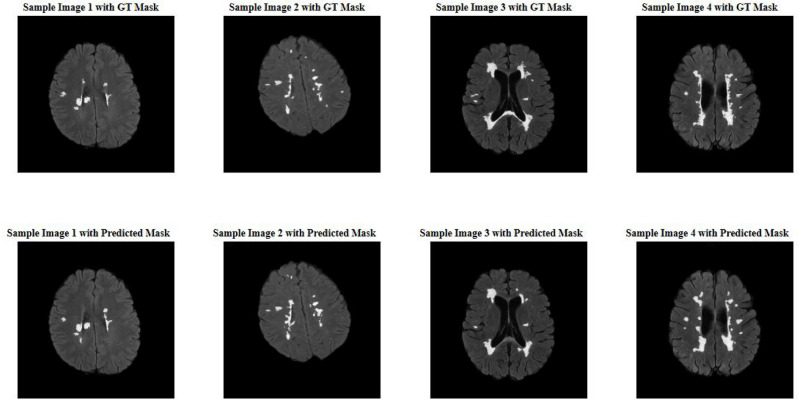
Example slices from the dataset in the axial plane, displaying actual labels (top row) and corresponding predicted labels by the network (bottom row).

In Fig. 6, four examples of slices from the test set are depicted. The images in the first-row show slices in the axial plane, along with their actual labels. The corresponding predicted images by the network are shown in the second-row. It is evident that the model has achieved high accuracy in identifying regions of plaques in brain tissue.

The proposed CNN-based system demonstrated significant improvements in the automatic segmentation of FLAIR MRI images for MS diagnosis. By effectively leveraging convolutional neural networks, the system achieved enhanced accuracy and reliability in segmenting both active and non-active lesions (plaques) specific to multiple sclerosis. These results highlight the system’s ability to provide detailed and precise segmentation, thereby advancing the capability to diagnose and monitor MS-related abnormalities more effectively through MRI imaging.

## Discussion

In this study, we developed and evaluated a CNN-based system for the automatic segmentation of FLAIR MRI images aimed at enhancing the diagnosis of multiple sclerosis (MS). Our results demonstrate significant advancements in both slice-level classification and voxel-level segmentation tasks.

### Slice-level classification

Our CNN model achieved promising results in slice-level classification, accurately distinguishing between MS-positive and MS-negative scans. For the internal testing set (center 1), the model achieved an accuracy of 83%, sensitivity of 100%, PPV of 75%, and NPV of 99%. Similarly, for the external testing set (center 2), the model achieved an accuracy of 76%, sensitivity of 100%, PPV of 68%, and NPV of 100%. These findings indicate robust performance in identifying MS-specific abnormalities across different datasets.

### Voxel-level segmentation

The CNN-based segmentation model demonstrated substantial improvements in voxel-level segmentation of MS lesions. In the internal testing set (center 1), the model achieved average values of 70% for DSC, 75.3% for PPV, 56% for intersection over union (IoU), 71% for sensitivity, and 28.3 for HD5. For the external testing set (center 2), corresponding values were 75% for DSC, 89% for PPV, 61% for IoU, 68.1% for sensitivity, and 31.5 for HD5. These metrics underscore the model’s effectiveness in accurately delineating MS lesions from brain tissue in MRI scans, highlighting its potential clinical utility.

### Impact of data augmentation

Data augmentation played a crucial role in improving model generalization and mitigating overfitting. By artificially expanding the training dataset with augmented images, we observed a reduction in validation loss and enhanced performance across both classification and segmentation tasks. This approach ensured that the CNN model learned robust features and patterns essential for accurate MS lesion detection without being overly sensitive to variations in input data.

### Clinical implications

The enhanced accuracy and reliability of our CNN-based segmentation system have significant clinical implications for multiple sclerosis (MS) management. Accurate segmentation of MS lesions supports clinicians in timely diagnosis, treatment planning, and disease monitoring. The ability to differentiate between active and non-active lesions is particularly valuable, providing crucial insights into disease progression and response to therapy. This capability facilitates the development of personalized patient management strategies tailored to individual disease dynamics.

### Future directions

Differentiating between active and non-active lesions represents a critical area for future investigation. While our study demonstrates the feasibility of segmenting MS lesions, further research is warranted to enhance the model’s capability in distinguishing lesion types based on dynamic imaging features. Future studies will explore advanced imaging modalities and longitudinal data analysis to improve sensitivity to lesion activity and chronicity. These advancements aim to enhance the clinical utility of our segmentation model in personalized MS management strategies.

While our model demonstrates high accuracy, it is important to note that variations in MRI acquisition protocols across different centers may affect the generalizability of the model. Factors such as differences in field strength, slice thickness, or scanner manufacturers could introduce variability in image quality and lesion visibility, potentially impacting segmentation performance. Future work should include testing on datasets from multiple centers to ensure robustness against such variations.

### Limitations

Despite the promising results, several limitations need consideration. The performance of the CNN model may vary depending on dataset diversity and size used for training and testing. Further validation on larger and more diverse cohorts is essential to assess generalizability across different clinical settings and populations. Integration of multimodal imaging data and longitudinal studies could bolster the model’s robustness and expand its clinical applicability.

## Conclusion

In conclusion, our study demonstrates that CNN-based segmentation of FLAIR MRI images is a promising approach for enhancing MS diagnosis and lesion characterization. The developed system shows considerable improvements in both accuracy and efficiency, paving the way for more reliable clinical decision-making in MS management. Future research efforts should focus on refining the model’s performance through collaborative efforts and large-scale validation studies, ultimately aiming to translate these advancements into routine clinical practice.

Future research should focus on integrating multi-modal MRI data, such as T1-weighted and T2-weighted images, to further improve segmentation accuracy and provide a more comprehensive assessment of MS lesions. Additionally, exploring the application of our model in clinical settings, such as real-time diagnostic workflows or longitudinal monitoring of disease progression, could validate its practical utility and impact on patient care.

## Supplementary Information

Supplementary Information.

## Data Availability

The datasets generated and analyzed during the current study are not publicly available due to patient privacy concerns but are available from the corresponding author on reasonable request.
